# Giant Faraday Rotation in Metal-Fluoride Nanogranular Films

**DOI:** 10.1038/s41598-018-23128-5

**Published:** 2018-03-21

**Authors:** N. Kobayashi, K. Ikeda, Bo Gu, S. Takahashi, H. Masumoto, S. Maekawa

**Affiliations:** 1 Research Institute for Electromagnetic Materials, Tomiya, 981-3341 Japan; 2 Advanced Science Research Center, Japan Atomic Energy Agency, Tokai, 319-1195 Japan; 3 Institute for Materials Research, Tohoku University, Sendai, 980-8577 Japan; 4 Frontier Research Institute for Interdisciplinary Sciences, Tohoku University, Sendai, 980-8578 Japan

## Abstract

Magneto-optical Faraday effect is widely applied in optical devices and is indispensable for optical communications and advanced information technology. However, the bismuth garnet Bi-YIG is only the Faraday material since 1972. Here we introduce (Fe, FeCo)-(Al-,Y-fluoride) nanogranular films exhibiting giant Faraday effect, 40 times larger than Bi-YIG. These films have a nanocomposite structure, in which nanometer-sized Fe, FeCo ferromagnetic granules are dispersed in a Al,Y-fluoride matrix.

## Introduction

**Figure 1:**
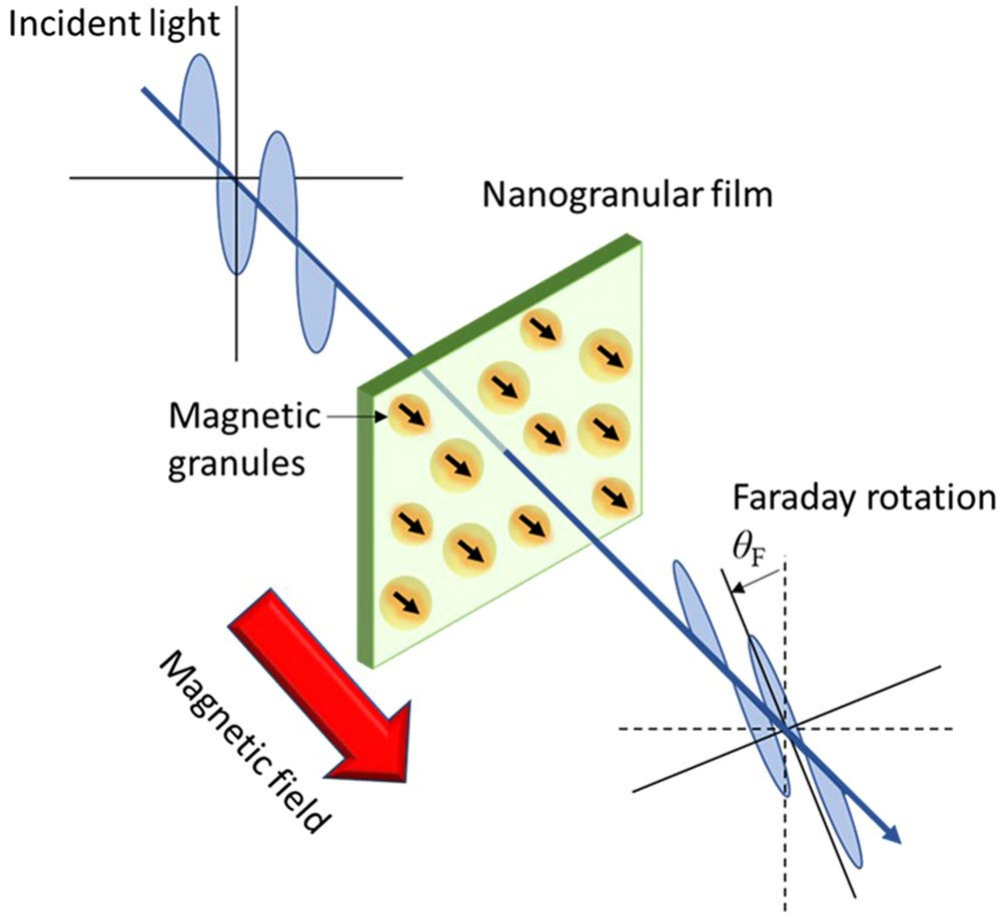
Image of Faraday effect. The Faraday effect causes rotation of the plane of polarization of an incident light by changing the magnetic field. Faraday material with a large Faraday rotation angle makes it possible to miniaturize the device.

Optical isolators utilizing Faraday rotation are of importance in many applications, such as quality assurance of optical amplifiers^1^, optical ring lasers^2^, and optical communication systems^3–5^. They are highly reliable and important tools to support advanced information technology. An image of Faraday effect is shown in Fig. 1. However, since the discovery of the bismuth garnet Bi-YIG^6,7^ in 1972, no material with Faraday effect beyond Bi-YIG has been found. Although thin films with a large Faraday angle have been examined to miniaturize optical devices, their properties are inferior to bulk Bi-YIG^8,9^. Therefore, optical devices are very limited^10,11^.

Here we introduce (Fe, FeCo)-(Al-, Y-fluoride) nanogranular films exhibiting Faraday rotation 40 times larger than that of Bi-YIG at the wavelength of optical communication band (1500 nm). These films have a nanocomposite structure, in which nanometer-sized Fe or FeCo ferromagnetic granules are dispersed in an Al-,Y-fluoride matrix. Nanogranular materials consisting of nanometer-sized magnetic metal granules and a ceramic insulating matrix exhibit various functional properties depending on the composition ratio of the two elements, granules and matrix^12–15^. In addition, since they are easily fabricated and are thermally stable, they have significant practical advantages. For example, the films have been applied in magnetic sensors^16,17^. A new transparent ferromagnetic material, FeCo-(Al-fluoride) nanogranular structure with a new magneto-optical effect, was recently proposed^18^. The material is transparent enough even for short wavelengths of light (less than 400 nm) and exhibits 90% transmittance at a wavelength of 1500 nm. Magnetization exceeds 18 kA/m (0.023 T) at room temperature.

## Results

### Faraday effect and structure of (Fe,FeCo)-(Al-fluoride) films

**Figure 2:**
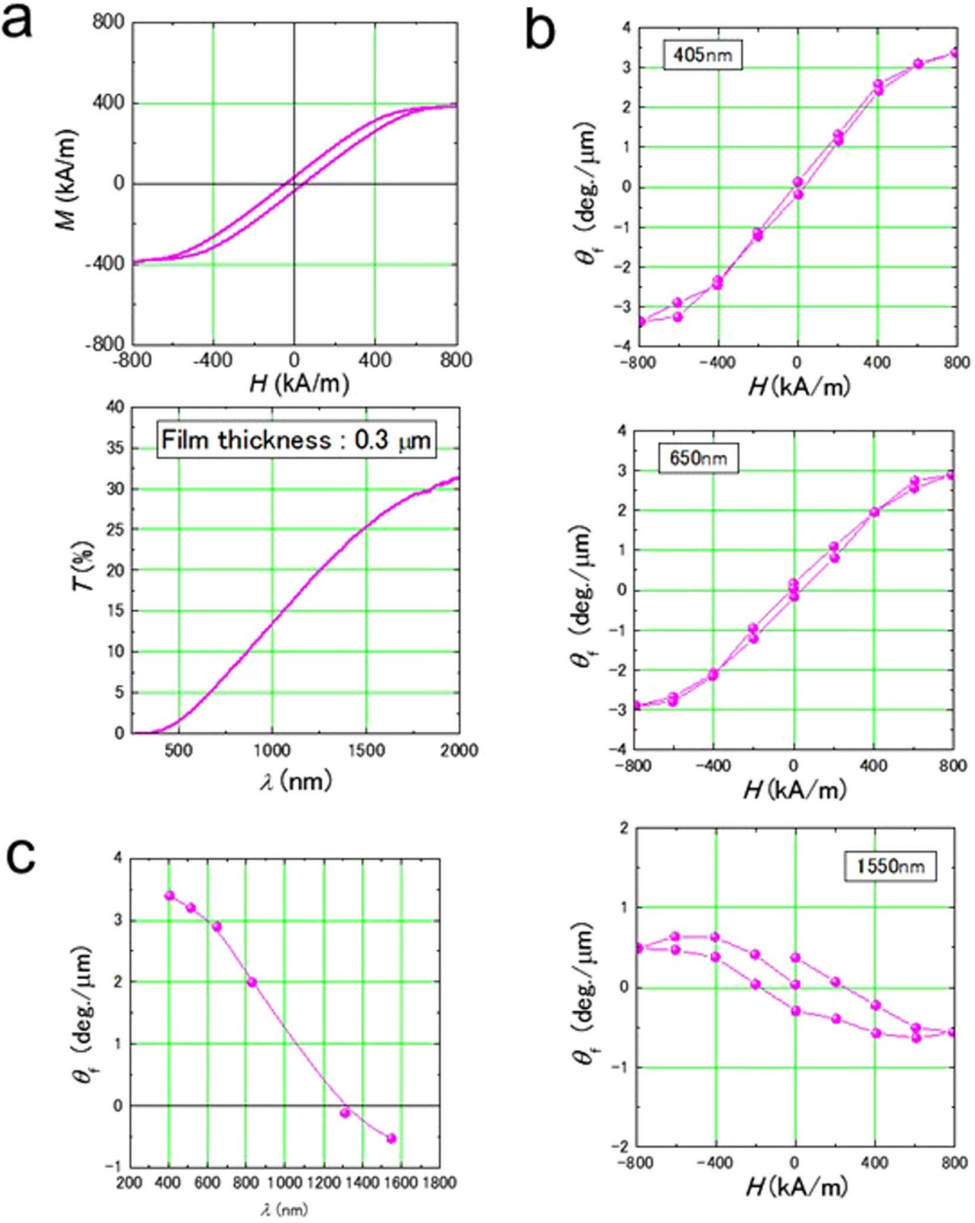
Faraday effect of Fe_26_Al_28_F_46_ film. (**a**) Magnetization curve and optical transmittance of Fe_26_Al_28_F_46_ film (0.3 μm thick) deposited on the 660 °C substrate. The magnetization curve was measured by applying a magnetic field perpendicular to the film surface. This direction is the same as in Faraday effect measurement. The magnetization curve exhibits hysteresis, indicating that the film is ferromagnetic. The measurement of transmittance is in the wavelength range of ultraviolet (400 nm) to near infrared (2000 nm) by Fourier transform infrared spectroscopy (FTIR). Using FTIR, only film transmittance was measured with reference to the substrate glass at the same time. (**b**) Faraday loops of Fe_26_Al_28_F_46_. The Faraday rotation angle is plus at wavelengths 405 and 650 nm and minus at 1550 nm. (**c**) Relationship between the wavelength of incident light and the Faraday rotation angle. The Faraday rotation angle changes the sign around a wavelength of 1200 nm.

Figure 2a presents the magnetization curve and optical transmittance of a Fe_26_Al_28_F_46_ film deposited on a glass substrate heated to 660 °C. Magnetization exceeds 387 kA/m (0.49 T) at room temperature. The film is ferromagnetic and transparent. The Faraday loop of this film is depicted in Fig. 2b. The loop was measured with incident light wavelengths of 405, 650 and 1550 nm. The magnetization and the Faraday loop exhibit the same behavior. The Faraday loop at 1550 nm is opposite from those at 405 and 650 nm. Figure 2c shows the relationship between wavelength and Faraday rotation angle. The angle changes from plus to minus around 1200 nm, and the value is 3.4 deg./μm at 405 nm and −0.53 deg./μm at 1550 nm.

**Figure 3:**
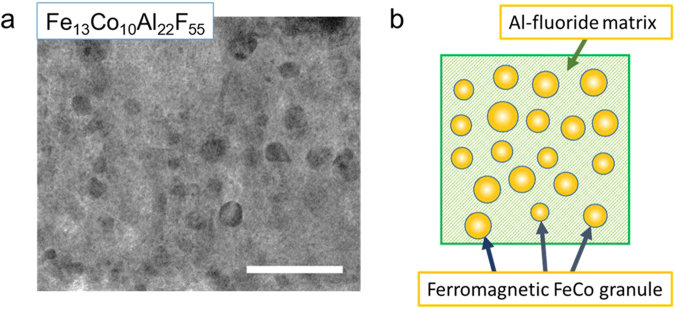
Nanogranular structure of FeCo-(Al-fluoride) film. (**a**) High-resolution transmission electron microscope image obtained from Fe_13_Co_10_Al_22_F_55_ film. (scale bar: 100 nm) (**b**) Schematic of nanogranular film with nanometer-sized granules dispersed in an insulator matrix. FeCo alloy granules with diameters exceeding 10 nm exhibit ferromagnetism because the granules are larger than the superparamagnetic critical diameter at room temperature.

Figure 3a presents the high-resolution transmission electron microscope image obtained from the Fe_13_Co_10_Al_22_F_55_ film deposited on the substrate at 600 °C. The film consists of FeCo magnetic alloy of nanometer-sized granules dispersed in an Al-fluoride matrix. The micrograph has many dark circles with diameters ranging from 10 to 15 nm. In addition, the bright section covers the whole area. The dark circles correspond to FeCo alloy granules, and the bright section indicates the Al-fluoride matrix. The schematic of the nanogranular structure is illustrated in Fig. 3b. When deposited on a heating substrate, the FeCo alloy granules become larger. FeCo granules with diameters 10–20 nm are single domain^19^ and exhibit ferromagnetism because the granules are larger than the superparamagnetic critical diameter at room temperature^20^.

### Giant Faraday effect of nanogranular films

**Figure 4:**
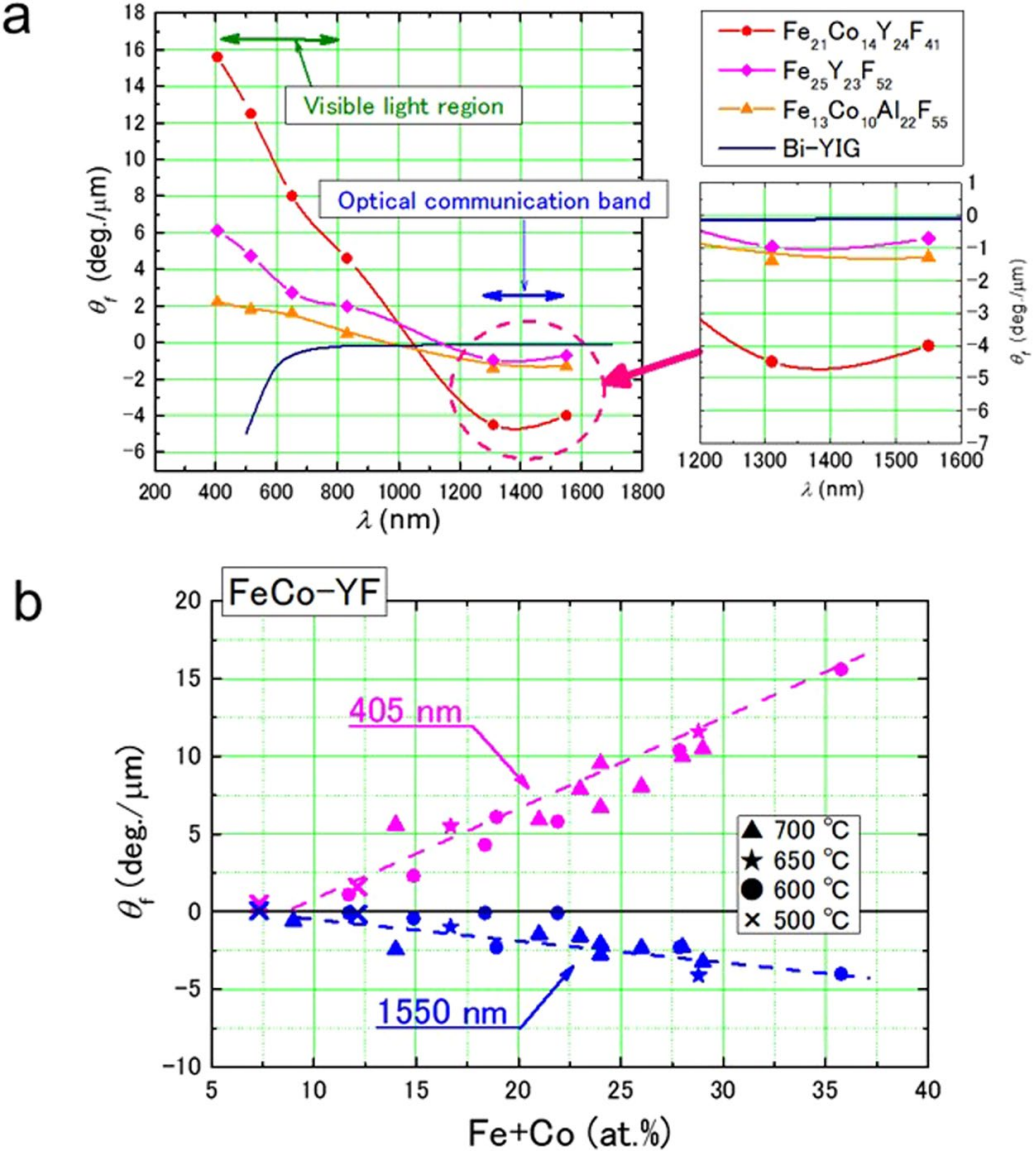
Giant Faraday effect in nanogranular films. (**a**) Relationship between the wavelength of incident light and the Faraday rotation angle of Fe_21_Co_14_Y_24_F_41_, Fe_25_Y_23_F_52_ and Fe_13_Co_10_Al_22_F_55_ films. The films were deposited on substrates of 600 °C, 550 °C and 680 °C. At these temperatures, each film is ferromagnetic. The value of Bi-YIG, which is a practical manufacturing material is also indicated. (**b**) Dependence of the Faraday rotation angle on the Fe + Co concentration of FeCo-(Y-fluoride) films at wavelength of 405 and 1550 nm. These films are deposited on heating substrate(500 to 700 °C).

Wavelength dependence of the Faraday rotation angle of Fe_21_Co_14_Y_24_F_41_, Fe_25_Y_23_F_52_ and Fe_13_Co_10_Al_22_F_55_ are presented in Fig. 4a, along with that of bulk Bi-YIG^8^ for comparison. All the nanogranular films depicted in Fig. 4a have much larger absolute Faraday rotation angles than Bi-YIG. It is noteworthy that the value of the Faraday rotation angle of Bi-YIG decreases with increasing wavelength. The angle in the wavelength of 1310 to 1550 nm, which corresponds to the bands of optical communication, is small in Bi-YIG. In contrast, the nanogranular films exhibit large values in optical communication bands.

Figure 4b indicates the Faraday rotation angles at wavelengths of 405 and 1550 nm as functions of the Fe + Co content in films deposited on substrates of different temperatures. The Faraday rotation angle increases with the Fe + Co content, indicating that the Faraday effect of the nanogranular films is due to the magnetic state of the FeCo granules. In this study, the substrates were heated to give ferromagnetism to the films by controlling the granular size. Depending on the substrate temperature, granular size may change; however, neither ferromagnetic properties nor Faraday rotation angle are affected.

Table 1 lists the Faraday rotation angles in the visible light region (650 nm) and the optical communication band (1550 nm) of the nanogranular films, as well as that of Bi-YIG for comparison. At both wavelengths, the Faraday rotation angles of the nanogranular films are much larger than that of Bi-YIG; for example, the angle of the Fe_21_Co_14_Y_24_F_41_ film is about 40 times larger than that of Bi-YIG at 1550 nm. This is a great practical advantage. In addition, the nanogranular materials are thin films (less than 1 μm thick). In contrast, Bi-YIG is a bulk material (1 mm thick). Optical devices can be miniaturized and integrated using the thin-film materials.

**Table 1:** Faraday rotation angles. Faraday rotation angle of Fe_26_Al_28_F_46_, Fe_13_Co_10_Al_22_F_55_, Fe_25_Y_23_F_52_, and Fe_21_Co_14_Y_24_F_41_ films, and of Bi-YIG bulk at light wavelength of 650 and 1500 nm.

material	*θ*_*f*_(deg./*μ*m)	
	*λ* = 650 nm	λ = 1550 nm
Fe_26_Al_28_F_46_	2.9	−0.53
Fe_13_Co_10_Al_22_F_55_	1.6	−1.3
Fe_25_Y_23_F_52_	2.7	−0.71
Fe_21_Co_14_Y_24_F_41_	8.0	−4.0
Bi-YIG	−0.6	−0.11

Nanogranular Fe,FeCo-(Al-,Y-fluoride) films are new Faraday materials. They exhibit much larger Faraday rotation angles than Bi-YIG. The angle is 8.0 deg./μm in the visible light region (650 nm) and −4.0 deg./μm in the optical communication band (1550 nm) in the Fe_21_Co_14_Y_24_F_41_ film. This value at 1550 nm is 40 times larger than that of Bi-YIG. The Faraday rotation angle of the nanogranular films depends on the wavelength on incident light, and the sign changes at 1000 to 1200 nm.

### Mechanism of giant Faraday effects in nanogranular films

First, we calculated the electronic states at Fe/insulator interfaces in density functional theory to clarify the mechanism of giant Faraday effect. In the density functional theory calculation with the WIEN2K package^21^, we obtain the occupation number *n*_d_ of 3d orbitals of Fe atoms, spin moment *S*^*z*^, and orbital moment *L*^*z*^ in Fe-bcc (body-centered cubic) bulk, Fe(001) surface, Fe-2ML with ML being the monolayer, and Fe/insulator interfaces with the insulators being MgO, AlF_3_ and YF_3_.

Generalized gradient approximation (GGA) is used with spin-orbit (SO) coupling in the calculation. For bulk Fe and Fe(001) surface, our GGA + SO results are in good agreement with previous calculations^22,23^. It is clear that the orbital moment of the Fe atom is enhanced at the Fe(001) surface, Fe-2ML, and Fe/insulator interfaces compared with that of bulk.

**Figure 5:**
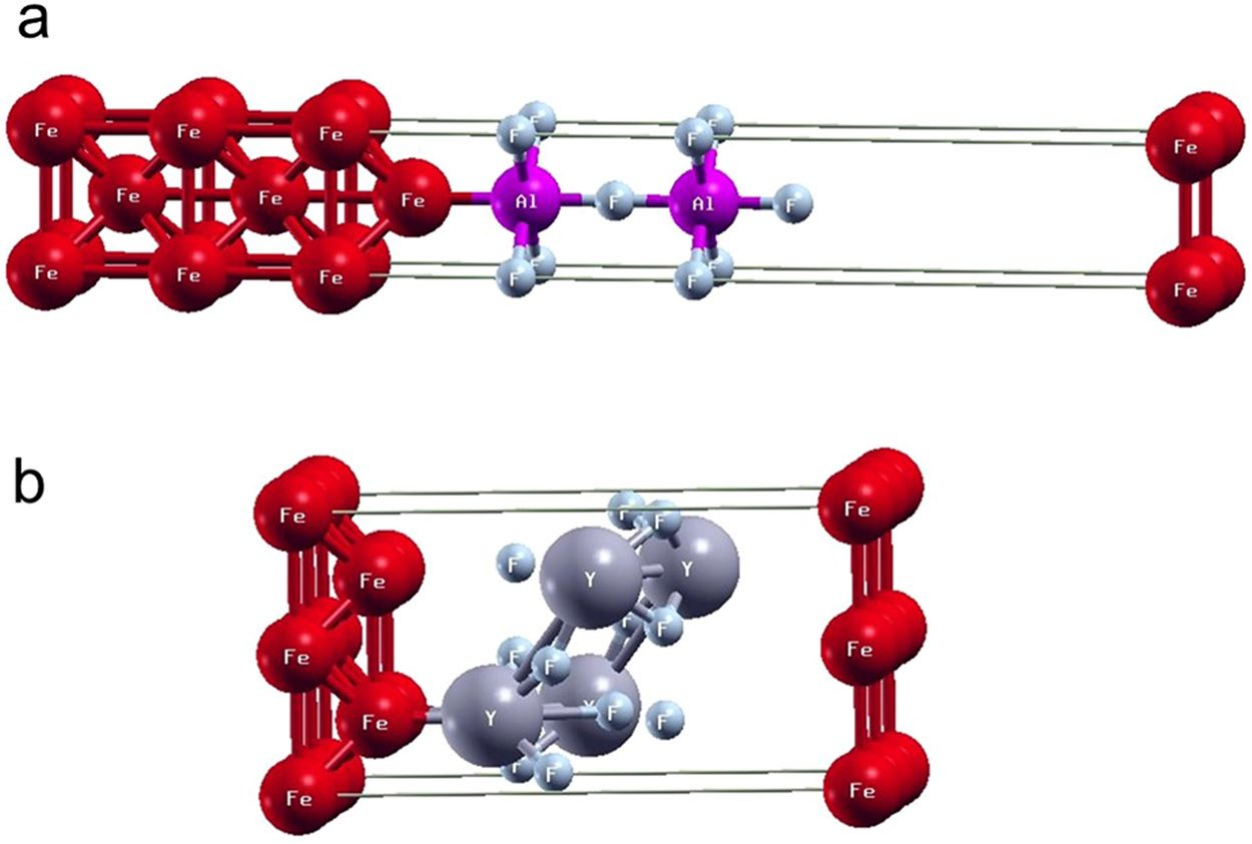
Model of Supercells. Supercells of (**a**) Fe/AlF_3_ and (**b**) Fe/YF_3_ interfaces used in the density functional theory calculation.

Fe (Table 2). The supercells of Fe/AlF_3_ and Fe/YF_3_ interfaces used in the calculation are depicted in Fig. 5. Enhancement of the orbital moment obtained on 3d transition metal surfaces is similar to that in previous studies^22,23^. It has been argued that the enhanced orbital moment comes from the reduced coordination of surface atoms, which causes narrower 3d electron bands and higher density of states at the Fermi level^23^. We use a similar argument for Fe-2ML and Fe/insulator interfaces: the Fe atoms at the interfaces have low-dimensional nature, which causes localized 3d electrons.

**Table 2:** Calculated results of occupation number *n*_d_, spin moment *S*^z^, and orbital moment *L*^z^. For 3d orbitals of the Fe atom, density functional theory calculation results of occupation number *n*_d_, spin moment *S*^*z*^, and orbital moment *L*^*z*^ in Fe-bcc bulk, Fe(001) surface, Fe-2ML with ML being the monolayer, and Fe/insulator interfaces with insulators being AlF_3_ and YF_3_. In this calculation, we use GGA and include SO coupling.

Systems	Occupation *n*_d_	Spin *S*^*z*^ (μ_B_)	Orbit *L*^*z*^ (μ_B_)
Fe-bcc bulk	5.96	2.27	0.041
Fe (001) surface	5.95	2.96	0.091
Fe-2ML (monolayer)	6.02	2.93	0.076
Fe/AlF_3_ interface	6.07	2.12	0.142
Fe/YF_3_ interface	5.53	3.22	0.065

Our results suggest that the orbital moment is enhanced in various interfaces and is almost independent of the interfaces. Since the electronic states including the orbital moment are similar between interfaces, in the following Maxwell-Garnett theory the dielectric tensor ${\hat{{\epsilon }}}_{m}(\lambda )$ of magnetic nanogranules is calculated using the density functional theory in the supercell of Fe-2ML as a function of light wavelength *λ*.

Next, we calculated Faraday rotation angle using the Maxwell-Garnett theory. The Faraday effect is a magneto-optical phenomenon caused by interaction between light and magnetic moment and gives rise to the rotation of light-polarization when a polarized light is transmitted through a magnetic film. The angle of rotation is the Faraday rotation angle given by^24^

1 $${\theta }_{f}(\lambda )=\frac{\pi d}{\lambda }{\rm{Im}}[\frac{{{\epsilon }}^{xy}(\lambda )}{{\varepsilon }^{xx}(\lambda )}],$$

where *ϵ*^*xx*^ and *ϵ*^*xy*^ are the diagonal and off-diagonal components of the dielectric tensor, *d* is the thickness of the magnetic film and *λ* is the light wavelength. The film thickness in Eq. (1) is fixed to *d* = 1 μm. In the Maxwell-Garnett theory^25^ for magnetic nanogranular films with spherical nanogranules dispersed in an insulator matrix, the effective dielectric tensor is given by^26–29^

2 $$\hat{{\epsilon }}(\lambda )={\hat{{\epsilon }}}_{d}+3{p}_{m}({\hat{{\epsilon }}}_{m}(\lambda )-{\hat{{\epsilon }}}_{d})\frac{{\hat{{\epsilon }}}_{d}}{(1-{p}_{m}){\hat{{\epsilon }}}_{m}(\lambda )+(2+{p}_{m}){\hat{{\epsilon }}}_{d}},$$

where ${\hat{{\epsilon }}}_{d}$ and ${\hat{{\epsilon }}}_{m}(\lambda )$ are the dielectric tensors of the insulator matrix and magnetic nanogranules, and *p*_*m*_ (0 ≤ *p*_*m*_ ≤ 1) is the volume fraction of magnetic nanogranules. The Maxwell-Garnett theory is applicable at small *p*_*m*_, since it is assumed that nanogranules are spatially separated. Here, we consider ferromagnetic nanogranules of Fe in a nanogranular film. The values of ${{\epsilon }}_{d}^{xx}=2$ and ${{\epsilon }}_{d}^{xy}=0$ are used for the insulator matrix. For *p*_*m*_ = 1, $\hat{{\epsilon }}(\lambda )$ is reduced to ${\hat{{\epsilon }}}_{m}(\lambda )$ of pure Fe.

**Figure 6:**
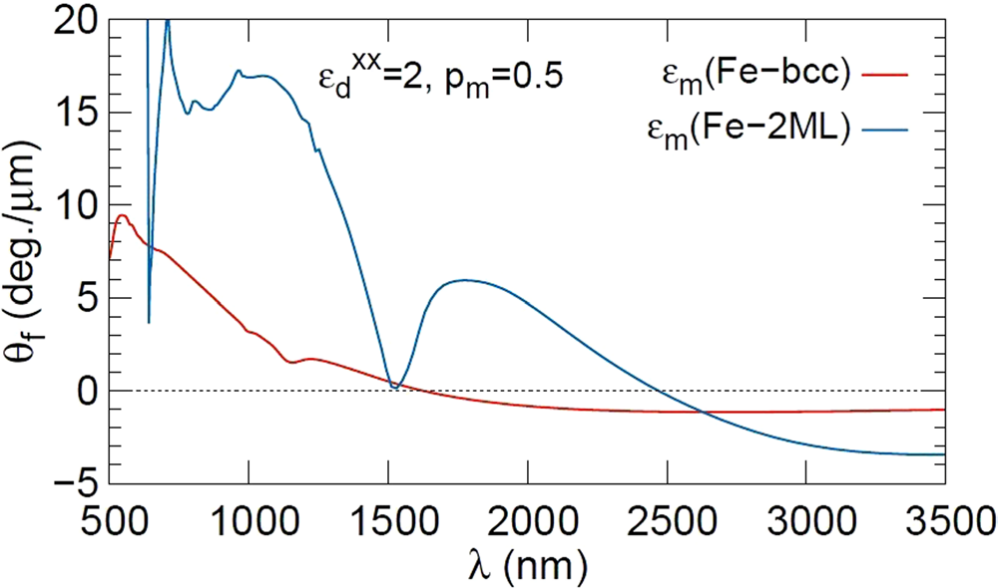
Calculated result of Faraday effect. Faraday rotation angle *θ*_*f*_ of granular films vs. light wavelength *λ* calculated in the effective dielectric tensor (Eq. (2)) of the Maxwell-Garnett theory. The parameters ${{\epsilon }}_{d}^{xx}=2$, *p*_*m*_* = *0.5 and *d = *1 μm are used. The angles *θ*_*f*_ with dielectric tensors ${\hat{{\epsilon }}}_{m}(\lambda )$ of Fe-2ML are denoted by blue lines, and those of Fe-bcc bulk are denoted by red lines.

For a nanogranular film, it is difficult to obtain ${\hat{{\epsilon }}}_{m}(\lambda )$ by taking into account the modification of electronic structure of granules near the interface. Thus, we assume that ${\hat{{\epsilon }}}_{m}(\lambda )$ is described by the dielectric tensor of Fe-2ML, where the supercell consists of 2-ML Fe and 2-ML vacuum and interfacial Fe atoms. Using ${\hat{{\epsilon }}}_{m}(\lambda )$ of Fe-2ML, we evaluate $\hat{{\epsilon }}(\lambda )$ in Eq. (2) and obtain *θ*_*f*_. The result for *p*_*m*_ = 0.5 is denoted by the blue line in Fig. 6. We find that the sign of *θ*_*f*_ changes at long wavelengths *λ* of 2500 nm.

We also assume that ${\hat{{\epsilon }}}_{m}(\lambda )$ is described by the dielectric tensor of Fe bcc bulk numerically obtained in density functional theory calculation with the Quantum-Espresso package^30^. The result for *p*_*m*_ = 0.5 is denoted by the red line in Fig. 6. Sign change occurs at a long wavelength of *λ* of 1500 nm.

In Fig. 6, it is clear that the magnitude of the Faraday rotation angle *θ*_*f*_ for Fe-2ML is larger than that for Fe-bcc in the whole wavelength range. This result indicates that the larger orbital moment in Fe-2ML gives rises to a larger magnitude of *θ*_*f*_. This result is in qualitative agreement with experimental results for nanogranular films. We note that the electronic states including orbital moment are similar between interfaces as discussed before.

As a result, we conclude that the enhancement of the Faraday effect in magnetic nanogranular films originates from the contribution of interfacial magnetic atoms.

## Discussion

We analyzed the mechanism of the giant Faraday angle in nanogranular films. In each nanogranule, the surface/interface produces a main contribution to physical properties, where the orbital magnetic moments of 3d electrons are enhanced^31^. Orbital magnetic moments are of crucial importance for the Faraday effect, since the off-diagonal component of the dielectric tensor is given by spin-orbit coupling. Using the Maxwell-Garnett theory^25^, we examined the Faraday rotation angle as a function of the wavelength of light in nanogranular films with 50% volume fraction of Fe granules with dielectric tensors of bulk-Fe and 2 mono-layer-Fe. Here, in 2 mono-layer-Fe, all Fe atoms correspond to surface ones. We found that in nanogranular films with a dielectric tensor of 2-mono-layer-Fe, the Faraday rotation angle is enhanced in the short wavelength region and exhibits a sign change in the long wavelength region. These results explain the experimental data.

Giant Faraday materials were discovered for the first time in 45 years. These materials will contribute greatly to the miniaturization and integration of optical devices.

## Methods

### Preparation of thin film samples

Thin films were prepared using tandem deposition^32^ with a conventional RF-sputtering apparatus. The films were 0.3 to 1.0 μm thick. Sputter deposition was performed on a 50 × 50 mm glass substrate (Corning Eagle 2000) at 500 to 700 °C in an argon atmosphere with 1.3 Pa pressure during deposition, using a 76 mm-diameter Fe or Fe_60_Co_40_ alloy disk as the metal target and a 76 mm-diameter AlF_3_ or YF_3_ powder compacted disk as the insulator ceramics target.

### Composition and structural analysis

The composition ratio of Fe,Fe-Co (granule) and Al-,Y-F (matrix) was controlled by changing the RF power applied to each target. The chemical composition of Fe, Co, Al, Y and F in the thin films was analyzed using wavelength dispersion spectroscopy (WDS). For structural analysis, transmission electron microscopy (TEM) was performed on several selected thin films.

### Measurements of magnetic properties, optical transmittance and Faraday angle

The magnetization curves were measured using an alternating gradient force magnetometer (AGM). Optical transmittance was measured using Fourier transform infrared spectroscopy (FTIR) with a measurement waveband of 400 to 2000 nm. The incident light is transmitted through the film samples or absorbed. The Faraday angle was measured using six laser light sources (405, 515, 650, 830, 1310 and 1550 nm). The magnetic field was applied from 0 to ± 800 kA/m perpendicular to the film surface of the samples in measurement of magnetization curves and Faraday angle. All measurements were carried out at room temperature.
